# Critical Social Science in Sport Management Research: A Scoping Review

**DOI:** 10.3389/fspor.2022.812200

**Published:** 2022-01-28

**Authors:** Kerri Bodin, Georgia Teare, Marijke Taks

**Affiliations:** Faculty of Health Sciences, School of Human Kinetics, University of Ottawa, Ottawa, ON, Canada

**Keywords:** philosophical paradigms, research approach, state of the field, theoretical contribution, systematic reviewing

## Abstract

Sport management scholars have called for the application of broader research approaches, including critical social science. Such approaches help uncover the less-desirable aspects of sport and, therefore, offer a basis for positive change. While there have been advancements in the use of innovative research approaches over time, there remains little understanding of how these calls have been addressed. The purpose of this scoping review is to examine trends, gaps, and the use of critical social science and associated approaches in sport management scholarship. Two hundred sixteen relevant articles were identified through a database search (i.e., five platforms), complemented with a manual search of 419 journals. Results indicate that an increase in critical research published in sport management journals was evident following 2005. Findings suggest that there remains space for increased publication of critical social science work in sport management journals and for researchers to better articulate their research approaches in scholarly outputs.

## Introduction

In the past two decades, scholars have increasingly called for sport management researchers to include more critical social science in their work (Amis and Silk, 2005; Skinner and Edwards, 2005; Chalip, 2006). Notably, in Frisby's (2005) Zeigler Award address, a prestigious award offered by the North American Society of Sport Management (NASSM), the use and applicability of critical approaches to advance the sport management field was highlighted. Frisby (2005) stated that critical approaches to sport management research would help to illuminate the “ugly” sides of sport management in hopes of informing change to improve practice. Similarly, in the introduction to a special issue on critical reflection in sport management, Amis and Silk (2005) acknowledged that while sport management journals published a variety of research approaches, perspectives, and theories, there was still much to unpack regarding the philosophical and political backbone of work in the field. Critical approaches are heavily employed in sport sociology, and scholars have further suggested that drawing on critical sociological perspectives in sport management can strengthen the field and our understanding of power in sport organizations (Knoppers, 2015). Alongside such calls for the use of more diverse research approaches, scholars have recently employed a variety of paradigmatic, methodological, and theoretical strategies in their work (e.g., McSweeney and Faust, 2019). For this study, we focus on critical social science, which is “a way of empowering individuals by confronting injustices in order to promote social change” (Frisby, 2005, p. 2). This includes a variety of theories (e.g., critical theory; Kincheloe and McLaren, 2011) and associated research approaches (e.g., participatory action research). Critical research approaches, and articulating such approaches clearly, help to unpack systemic issues within social spheres, and therefore offer new insight into areas that need improvement. Critical approaches typically offer the opportunity to investigate power structures within taken-for-granted systems and provide researchers with the tools for understanding how and why systems may be exclusive, oppressive, or otherwise need improvement (Knoppers, 2015; Sveinson et al., 2021). In sport specifically, this is important for practice to determine how to best provide programming and sport resources to as many people as possible.

Research has expanded beyond post-positivist approaches and has begun to explore various lenses through which sport management topics can be understood. One of many possible examples includes Singer's (2005) call for the use of critical race theory to address issues of race and ethnicity in sport. More recently, Chen and Mason (2019) made similar suggestions for decentering colonial perspectives and employing settler colonialism in research. Critical realist approaches to policy and management have also been brought forth as ways to incorporate broader perspectives in sport research more generally, and therefore offering a way to advance social justice objectives (Downward, 2005). These critical paradigms have offered significant advancement in understanding different points of view and have added the value of different research perspectives in the field.

With the addition and increase in the use of critical, interpretive, and constructivist paradigms, qualitative research methodologies have also increased in use. However, there remains work to be done in advancing these methods (Singer et al., 2019). Methodological approaches such as autoethnography (Hoeber and Kerwin, 2013; Cooper et al., 2017) and participatory action research (Frisby et al., 2005; Shaw and Hoeber, 2016; Hoeber and Shaw, 2017) are particularly valuable for critical social science. Most recently, Sveinson et al. (2021) have advocated for increased use of critical discourse analysis as a theory, methodology, and analysis in sport management scholarship. These methods engage diverse perspectives with a particular focus on advancing equity within sport, an important component of critical social science and associated research approaches. Further, upon investigating additional ontological and epistemological approaches, Quatman and Chelladurai (2008) advanced social network analysis as an avenue through which to address Frisby's (2005) call for advancing critical social science and associated research approaches. Alongside the increase in qualitative methodologies, mixed methods have begun to gain traction in recent years, as researchers have acknowledged the benefits of drawing upon diverse research methods in individual projects (van der Roest et al., 2015). Theoretically, scholars have sought to expand existing sport management research practices by introducing new theory and integrating theory from other fields into the management space (e.g., Sotiriadou and Shilbury, 2010; Kitchin and Howe, 2013). Each of these trends have advanced understanding of sport studies and sport management and have offered enriched perspectives regarding how to improve diversity, inclusion, and equity in sport management practice through challenging dominant power relations (Cunningham and Fink, 2006), in part addressing concerns raised by various scholars and Zeigler award winners (e.g., DeSensi, 1994; Frisby, 2005; Chalip, 2006; Fink, 2016; McGarry, 2020). However, despite shifts in recent years to accept more diverse research perspectives and methodologies, the uptake of the recent calls for the use of critical social science and associated research approaches to sport management research, the use of innovative research methodologies, and to further center these approaches in the sport management literature has yet to be further understood (Cunningham and Fink, 2006; Knoppers, 2015; Singer et al., 2019).

There is little systematic work investigating the scope and patterns in methodology and theory used in relation to critical social science. As such, the purpose of this scoping review is to examine the trends, gaps in research approaches, and the state of use of critical social science in sport management scholarship. To widen the scope of this investigation, we go beyond the sport management journals specifically, and investigate sport management work regardless of the journal domain within which it is published. In doing so, we offer significant contributions to the sport management field by highlighting ways in which critical social science and research approaches have been used and ways that future research may employ such approaches. Investigating the use of critical approaches in sport management is necessary to understand the growth and applicability of such approaches over time and their contribution to the sport management body of literature to inform future critical directions in sport management research. The use of critical approaches in sport management work will help push the management field to expand our understanding of sport's role in society, how it may be an exclusionary space and to provide a strong theoretical basis for practical improvements by challenging power relations, such as drawing attention to equity and inclusion, and mitigating social injustice in the sport field (McGarry, 2020). As evidenced by recent sociopolitical events (e.g., rise of the Black Lives Matter movement) and the visible response in the sport-space (e.g., Colin Kaepernick kneeling during the United States national anthem; the WNBA and NBA integrating racial justice messaging in their leagues), it is clear that while sport can be an exclusionary space, it can also be a societal vehicle for change. Therefore, by understanding how critical approaches have been used in sport management, we can inform how these approaches can continue to be used to advance a social justice agenda within sport management, with the potential and intent to have implications more broadly. We acknowledge that this work may be done elsewhere in fields such as sport sociology, with which sport management scholarship shares many approaches, theories, and research contexts (Knoppers, 2015). However, for the purposes of this scoping review and based on calls for more critical research approaches in sport management specifically, we have focused our attention on the field of sport management as defined in the key terms section below. After defining the key terms central to this scoping review, we then present an overview of the scoping review methods employed. Subsequently we present the results and discussion, highlighting the state of the field and offer suggestions for future research.

### Key Terms

#### Critical Social Science

Critical social science is “a way of empowering individuals by confronting injustices in order to promote social change” (Frisby, 2005, p. 2). This definition encompasses a wide variety of research approaches, including various critical theories (e.g., critical race theory, critical disability theory, etc.), various methods (e.g., participatory methods), and investigations of power structures (Kincheloe and McLaren, 2011). Therefore, we consider critical social science as including critical research approaches and the use of critical theory, as noted above. Critical theory is understood as a philosophical approach which considers historical and social context, accepts subjectivity, and addresses power (Guba and Lincoln, 1994). For this scoping review, we narrowed the scope of the study to include research that specifically stated employing a critical theoretical lens or taking a critical approach. While other studies have sought to code articles based on paradigmatic assumptions and underpinnings as opposed to explicitly stated philosophical paradigms (see van der Roest et al., 2015), this was not possible due to the number of articles collected for this review. Further, given that paradigms are described as belief systems (Guba and Lincoln, 1994), and therefore can be personal, we did not feel it was appropriate to assume or ascribe a critical approach to a paper where one was not stated by the author(s).

#### Sport Management

Sport, and therefore sport management, can have a variety of meanings depending on the context of the discussion. For example, Coakley (2003) defined sport as “institutionalized competitive activities that involve vigorous physical exertion or the use of relatively complex physical skills by participants motivated by intrinsic and extrinsic rewards” (p. 21). Meanwhile, others have described sport much more broadly, encompassing all forms of physical activity and both casual and organized forms of participation (Council of Europe, 2001). Sport management itself has been broadly described as any combination of skills pertaining to planning, organizing, leading, and evaluating in the context of sport and physical activity (DeSensi et al., 1990). Given the wide array of accepted definitions for sport and sport management, for the purposes of this study, we adopted a definition of sport management adapted from the NASSM website and the Journal of Sport Management. Therefore, we define sport management as the coordination of the production and marketing of sport services and sport organizations, including sport management education (North American Society for Sport Management, n.d.).

#### Scoping Review

A scoping review assesses the nature and extent of research evidence in a replicable and rigorous way (Grant and Booth, 2009; Whittemore et al., 2014). Scoping reviews provide an overview of a particular line of inquiry, including the size of available literature, scope of studies, and highlights gaps in study designs and approaches (Grant and Booth, 2009). A scoping review is the most pertinent type of review to address this study's purpose because contrary to other types of reviews such as systematic reviews and meta-analyses, a scoping review does not appraise or synthesize the findings of the articles (Arksey and O'Malley, 2005; Grant and Booth, 2009). As such, due to the nature of scoping reviews and the scope of this paper, an appraisal of the quality of critical research outlined in the included papers will not be provided herein. Indeed, synthesizing findings would not be meaningful in the present investigation as the focus is on revealing the approaches and theories used, rather than the outcomes from a particular research topic.

## Methods

This scoping review was conducted following Arksey and O'Malley's (2005) five-step framework, including Teare and Taks' (2020) extension of the process. In addition to Arksey and O'Malley's (2005) five-steps (i.e., Identifying the research question; Identifying relevant studies; Study selection; Charting the data; Collating, summarizing, and reporting results), Teare and Taks (2020) suggested that the process of identifying articles to be included in scoping reviews should be comprised of a minimum of two systematic approaches to article identification (expanding Arksey and O'Malley's step two: identifying relevant studies). This suggestion is based on previous findings that two different systematic approaches to article identification (i.e., database search and systematic manual search) led to different pools of articles, and thus a more comprehensive final pool of articles (Teare and Taks, 2020). As such, the approach taken here also includes the executions of both a traditional database and systematic manual search.

As per step one of the scoping review framework the selection process was established based on the following research question: “what are the trends, gaps, and state of the use of critical social science in sport management scholarship?”

### Article Selection Process

Preliminary readings of related articles (e.g., Alvesson and Deetz, 2000; Amis and Silk, 2005; Frisby, 2005) served to identify search terms and inclusion criteria (step two). For this scoping review, inclusion criteria consisted of: (1) scholarly, peer-reviewed articles, available online, and written in English; (2) authors must have specifically stated employing a critical approach; (3) the research must fit within the definition of sport management previously provided. The database search took place in January 2020, with year limits in place from 1985 (when NASSM was founded) to 2019. The systematic manual search included all issues appearing in journals between 1985 and 2019.

#### Database Search

Databases were chosen based on the research question and their likelihood to contain relevant articles. Based on preliminary readings and in consultation with a research librarian, the following five databases were used to search for articles published between 1985 and 2019: ProQuest Social Sciences, ABI Inform, Business Source Complete, SPORTDiscus, Sport Medicine and Physical Education Index. The following key search words were used [critic^*^ NEAR/3 (theor^*^ OR approach^*^ OR scien^*^)] OR [critic^*^ NEAR/3 soci^*^ NEAR/3 (theor^*^ OR scien^*^)] AND (sport^*^) OR [sport^*^ NEAR/3 (coordination OR product^*^ OR market^*^ OR manag^*^ OR admin^*^)]. The asterisks mean that any combination of letters can appear after the specified word or part of the word appearing before the asterisks. The brackets mean that words must appear in the order that they are written. The “NEAR/3” is a search function that allows for the search to include instances where zero, one, two, or three words separate the two words between which the function appears.

This initial search revealed 1,521 total articles. Duplicates were then removed, leaving 665 articles to be searched in the first round of screening. Two rounds of screening took place to determine the articles to be included in the final pool of sources among two researchers independently from one another (step 3). First, using Covidence (a review management program; www.covidence.org), titles and abstracts were screened against the inclusion criteria (i.e., scholarly, peer-reviewed articles, available online, written in English; authors have specifically stated employing a critical approach; the research fits within the definition of sport management) and exclusion criteria (i.e., no explicit statement of a critical approach; not within the area of sport management). Articles that were deemed to fit with the inclusion criteria by both researchers were moved directly to the second round of full-text screening. Articles that were excluded by both researchers were immediately eliminated from the pool of articles. When the researchers disagreed, the articles were flagged, and both researchers met to discuss the titles and abstracts against the inclusion/exclusion criteria. Articles that were mutually agreed to be potentially relevant were moved to the second round of screening. After this first round of title and abstract screening, and discussing discrepancies, 152 articles were moved onto the second round of screening. The second round of screening involved the researchers independently reading the articles in full. Articles that were agreed upon to meet the inclusion criteria were immediately included in the final pool of articles, and discrepancies were again discussed. After the round of full text screening, the database search yielded 112 articles.

#### Systematic Manual Search

As per Teare and Taks (2020), there are three steps involved in conducting a systematic manual search: (1) selecting the top field-specific journals (as determined by impact factors), (2) screening all issues for relevant articles (similar two-round process as the data base search: abstract screening, followed by full text screening); and (3) examining the reference lists of the identified articles for additional relevant journals. The same process is then completed for the journals of the newly identified articles; that is, a full journal search of these new journals was performed until no new journals arose (Teare and Taks, 2020). Top field-specific journals are a good starting-point for the systematic manual search as relevant articles are likely to appear in these journals. However, the systematic manual search is certainly not limited to only the identified field as it allows for journals from a variety of additional domains to be uncovered (Teare and Taks, 2020). Due to the varying terminology in interdisciplinary topics such as those in sport management, the key words used in the database search are likely limited to those words that researchers are familiar with, potentially excluding additionally relevant articles (Teare and Taks, 2020). As such, the additional journals identified in the systematic manual search are useful in bridging this gap (Teare and Taks, 2020).

As the context of this study is sport management scholarship, the most relevant field is sport management. Thus, the top three journals in sport management as per impact factors as of 2019 include: Sport Management Review (SMR), Journal of Sport Management (JSM), and the European Sport Management Quarterly (ESMQ). Thus, these three journals were selected as entry point for the manual search. While the period for article selection was set between 1985 and 2019, it should be noted that the first issues of these journals were published in 1987 for JSM, 1998 for SMR, and 2001 for ESMQ^1^. All articles in all issues were examined using the inclusion and exclusion criteria. Titles, abstracts, and keywords comprised the first round of screening, followed by full text screening. Articles selected from these three base journals, led to six rounds of additional journal searches. In total, 419 journals were examined to reveal 166 articles in the systematic manual search.

#### Combining the Search Methods

The database search yielded 112 relevant articles, while the systematic manual search yielded 166 relevant articles, for a total of 278 identified articles. When combining the results of the two search methods, only 62 articles were identified through both searches. Thus, this scoping review included 216 total unique articles (50 articles unique to the database search; 104 articles unique to the systematic manual search; 62 articles identified through both search methods; Supplementary Material). The distinctive outcome between the database search and the manual search is important as it provides further support for the extension to the scoping review framework by adding a comprehensive manual journal search (Teare and Taks, 2020).

### Data Analysis

Based on preliminary readings of scoping reviews (e.g., Inoue et al., 2015; Dowling et al., 2018; Hansell et al., 2021), categories of information to extract from the articles were developed prior to extracting the data (i.e., step 4 in the scoping review framework; Arksey and O'Malley, 2005). An overview of the data extraction criteria is provided in Table 1. Following data extraction, analyses were run to determine the relationships between data categories. As per step five from Arksey and O'Malley's (2005) framework (i.e., reporting results), the following sections report on descriptive analysis and trends that were evident from the extracted data. Specifically, comments on publication evolution, theories used, sport management focus, methodological approaches, and theoretical outputs will be offered.

**Table 1:** Data extraction categories.

**Category**	**Description**
Title	Article title
Keywords	Article key words
Journal	Journal name
Year of Publication^*^	Year
Journal domain^*^	E.g., sociology, geography, leisure, etc.
Purpose	Study purpose
Theory^*^	E.g., feminist theory, critical race theory, etc.
**Sport management focus**	
Context^*^	E.g., country context, organizational context
Area^*^	E.g., management, marketing, etc.
**Methodological approaches**	
Methods^*^	E.g., qualitative, quantitative, mixed, conceptual
Study design^*^	E.g., longitudinal, case study, etc.
Instruments	E.g., interviews, document analysis, etc.
Type of data^*^	E.g., primary, secondary, mixed
Study population^*^	E.g., youth, college athletes, female administrators, etc.
Type of analysis^*^	E.g., thematic, discourse, etc.
**Outputs**	
Key findings	Were key findings offered
Key implications	Were practical implications offered
Theoretical implications^*^	Were theoretical implications offered
Future research	Suggestions for future research
Limitations	Were limitations offered

* *Are elaborated upon in the body of the text*.

## Results and Discussion

Results from the analyses indicate specific trends and gaps associated with the use of critical social science in the sport management field. Findings are outlined below.

### Evolution of Publications Using Critical Social Science

As stated above, a total of 216 articles were identified for this scoping review. At face-value, this might seem like a large number, putting into question if there is even an issue with the amount of critical social theory used in sport management research. When considering the number of publications per year, this is in fact a concerningly small number. For example, our search produced 15 articles published in 2019. When considering the top three sport management journals specifically, there were 134 articles published in total in 2019 (JSM = 47, ESMQ = 33, SMR = 54). Of those 134 articles, only 4 (<1%; i.e., Chen and Mason, 2019; Shaw, 2019; Warner, 2019; Zipp et al., 2019) took a critical approach as per our inclusion requirements for this study.

**Figure 1:**
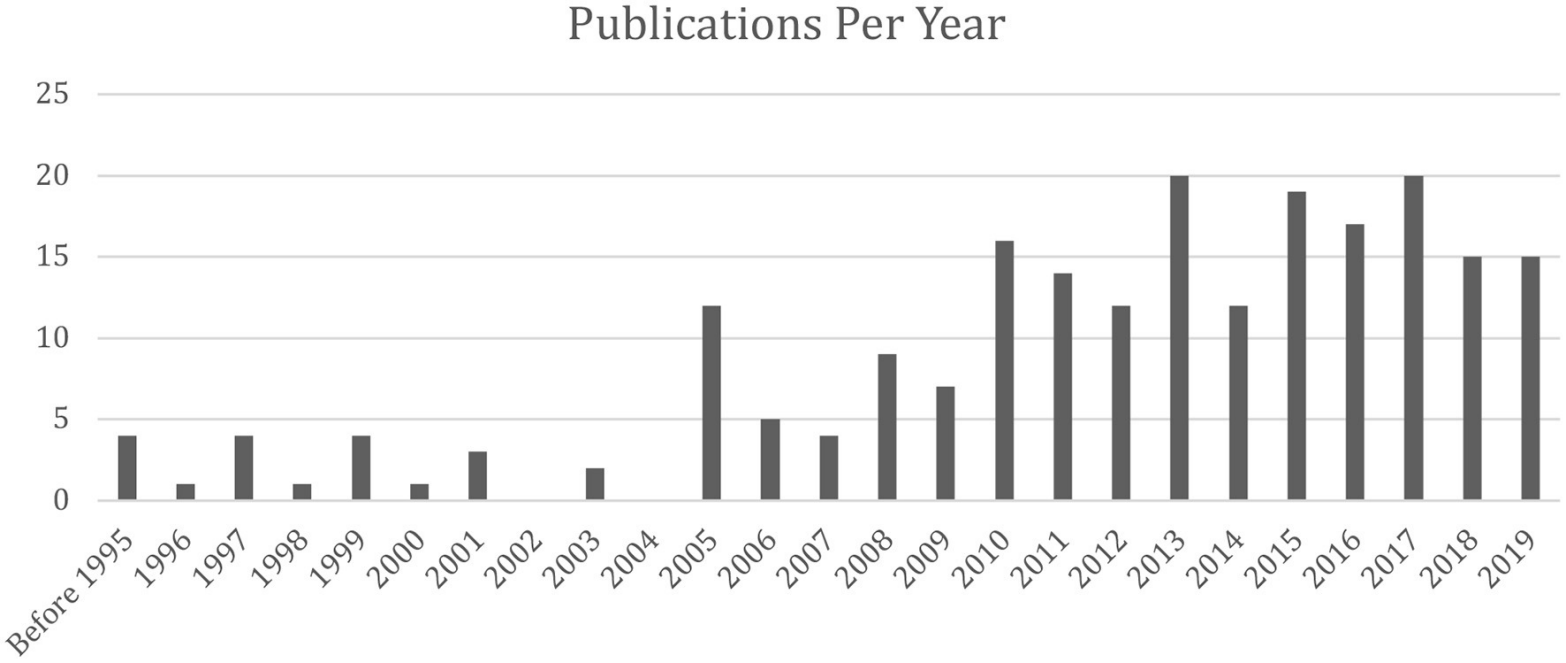
Critical social science publications in sport management per year (*N* = 216).

As seen in Figure 1, there had been an increase in the number of publications in 2005, followed by an increasing trend in publications per year. Perhaps Frisby's (2005) Zeigler lecture acted as a milestone for igniting the use of critical social theory in sport management studies, alongside other work published around the same time (e.g., Amis and Silk, 2005). Another possible explanation for the increasing number of publications using a critical social science approach is that an increasing number of total publications, regardless of paradigmatic approach, across social science and humanities domains has been documented in general (Engels et al., 2012), perhaps due in part to the increased use of technology, making article publication faster and less expensive for publishers, as well as more accessible to readers. With increasing publication numbers overall, there may be more opportunity for scholars who complete innovative methodological, theoretical, or paradigmatic work to get their research published.

#### Journal Domain

Not surprisingly, the largest proportion of studies published that used critical social science to address issues in sport management appear in journals that fall under the sport management and sport policy domains (*n* = 66; 31%; see Table 2). This finding is likely due to the framing of the scoping review itself, however what is noteworthy is the journal domains aside from sport management within which identified articles have been published. Sport sociology journals (*n* = 42; 19%) account for the next most popular outlets, followed by psychology/sociology (*n* = 21; 10%), physical activity and leisure (*n* = 20; 9%), education (*n* = 18; 8%), communication (*n* = 12; 6%), administration and business (*n* = 9; 4%), geography and public health (*n* = 8; 4%), and other (*n* = 20; 9%). These findings indicate that critical work is being conducted, however may end up being published outside of a sport management domain when considering the variation in journal outlets. The choice to publish in the non-sport-specific journals might stem from a desire (or requirement, e.g., tenure and promotion criteria) for scholars to publish in journals outside of sport for higher impact factors. The results regarding journals appearing in the general sociology/psychology category (e.g., American Behavioral Scientist, Disability and Society, Gender in Society, Journal of Sociology) might reflect this metrics-driven publishing phenomenon in academia. Although outside the scope of this study, it may be a fruitful avenue for future research to explore this idea further and to examine authors' decision-making processes regarding publishing outlets alongside editors' and associate editors' perceptions of their journals and fields.

**Table 2:** Number of annual critical social science publications in sport management by research domain and publication period (percentages between brackets).

**Year**	**Adm./bus**.	**Psych/Soc**	**Comm**.	**Educ**.	**Exercise/Leisure**	**Geography**	**Other**	**Sport Sociol**.	**Sport Management/Policy**	**Total *n* (%)**
Before 2004	1	0	0	3	4	1	0	4	7	20 (9)
2005–2009	2	3	4	3	5	0	2	2	15	36 (17)
2010–2014	3	7	4	3	4	4	8	19	22	74 (34)
2015–2019	3	11	4	9	7	3	10	17	22	86 (40)
Total *n* (%)	9 (4)	21 (10)	12 (6)	18 (8)	20 (9)	8 (4)	20 (9)	42 (19)	66 (31)	216 (100)

When further examining Table 2 for the yearly number of publications per journal domain, some interesting trends appear. The domains of Administration/business, Communication, Physical activity/leisure, and Geography have consistent contributions throughout the 35-year period examined. Though a consistently low number of publications appear in these domains, it indicates that there seems to be a niche for sport management topics with a critical lens in these domains. For example, corporate social responsibility is a dominant topic among the articles that appear in the administration and business domain (e.g., Giulianotti, 2015; Levermore and Moore, 2015). In the communication domain, many investigations highlight how sport media reinforced social inequity through subtle discourse (e.g., Buffington and Fraley, 2008; Lavelle, 2010; Ličen and Billings, 2013; Masucci and Butryn, 2013; Gill et al., 2017; Toffoletti, 2017). The geography domain contains many articles on the geographies of sport events (e.g., Levermore, 2011; Samatas, 2011; McGillivray et al., 2018). Interestingly, there are no common themes present in topics within the leisure domain, possibly because leisure studies are often rooted in critical perspectives.

In the education domain, there had been a consistent number of publications up until the 2015-2019 time-period, where the average number of publications more than doubled. This could be due to the two special issues in *Sport, Education, and Society* (i.e., Gender, physical education, and active lifestyles: contemporary challenges and new directions in 2018; Researching Education within Sport for Development in 2016) and *Quest* (i.e., Social Justice and Sport: Religious, Sociological, and Capability Perspectives in 2019) on critical themes in a sport context. Regardless, the publication of special issues on certain topics indicates a need for, and interest in, particular themes in research and therefore reflect trends in the field more broadly.

There is a consistently increasing trend in the number of publications in the psychology and sociology domains. When looking at the number of journals in the sport management domain, there is a clear spike in the number of publications from the “before 2005” timeframe to the 2005–2009 timeframe. This may be due in part to Frisby's (2005) call for the use of critical social theory in sport management research. This call could have inspired: (1) researchers to employ more critical social science and associated research approaches, and (2) journal editors and reviewers to accept more critical research for publication in sport management journals. Though the increasing trend continues into the following two timeframes, the increase is not as large, and seems to start to level–off. It is interesting to compare the sport management domain trends with that of the sport sociology domain. Sport management and sport sociology publications are quite similar in the “before 2005” timeframe. In the 2005–2009 timeframe, however, there is a decrease in publications in the sport sociology domain, further providing support for the openness of sport management journals to critical research. In the 2010–2014 and 2015–2019 timeframes, there was a large jump in the number of publications in the sport sociology domain, closing in on the number of publications in the sport management domain. This trend could indicate that the increase in the number of publications in the sport management journals could have been a “fad” in response to Frisby's (2005) Zeigler lecture, and there is less of an openness long-term for sport management journals to accept work using critical social science and associated approaches, and thus critical scholars in sport management are yet again choosing sport sociology journals as outlets for their work. As mentioned in the introduction, sport sociology and general sociology journals publish critical work extensively, and therefore authors may find their critical sport management research is more welcomed in those outlets compared to sport management or business/administration outlets. Additionally, scholars have pointed to the tendency of sport management journals to privilege post-positivist work over more critical or qualitative approaches (e.g., Knoppers, 2015; Sveinson et al., 2021). Analyzing future trends can provide insight into the accuracy of the assumption that sport management journals are overall less open to publishing critical research.

### Theory

A large proportion of studies (*n* = 54; 25%) included in this scoping review stated taking a “critical approach” but did not explicitly state they were using a critical theory or a critical paradigmatic position. A feminist approach (*n* = 45; 21%) accounts for the next largest proportion theories used in critical sport management research. Race-based theories (*n* = 36; 16%) are not far behind, followed by discourse analyses (*n* = 22; 10%), critical theory (*n* = 17; 8%), neoliberalism (*n* = 7; 3%), Settler and post-colonial theories (*n* = 6; 3%), (dis)ability theories (*n* = 4; 2%), Bourdieu's social theories (*n* = 4; 2%), and Other (*n* = 21; 10%). It is important to note that some studies used multiple theories. The most popular theory combination was a gender-based theory and a race-based theory (e.g., Travers, 2011; Prouse, 2015; Rankin-Wright et al., 2016), highlighting the importance of investigating sport through an intersectional lens (Shaw and Frisby, 2006; Anderson and McCormack, 2010).

Theory use is quite dispersed across the journal domains, with the following noteworthy observations. The largest proportion of race-based theory appears in the sport sociology domain (*n* = 10 of the 36 studies that use race-based theories), which is interesting because the largest proportion of overall studies are in the sport management domain. The same trend appears with critical theory (*n* = 8 of the 17 studies that use critical theory). Well over half of the settler/post-colonial studies appear in the sport management domain (*n* = 4 of the 6 studies that use a settler/post-colonial theory). Gender-based theories are the most frequently used in the sport management domain (*n* = 14 of the 45 studies that use a gender-based theory).

As discussed in the introduction and methods sections, it was necessary to clearly define inclusion criteria for the term “critical social science.” In limiting our scoping review to articles that explicitly stated the use of a critical theory or critical approach, the study at hand may have excluded articles that were underpinned by critical ways of thinking, addressed power relations, or could otherwise be considered “critical” work without having explicitly stated the approach. While this is indeed a limitation of the study, it was a necessary demarcation to ensure the scoping review was feasible and that assumptions regarding paradigmatic positioning of authors were avoided. Further, since we engaged in both a database and a manual search, we feel as though the articles included in the scoping review provide a thorough overview of the state of the field. Nonetheless, we realize that our search terms may have excluded some relevant work. Along these lines, our findings regarding the use and statement of theory suggest that there may at times be a lapse in communicating philosophical paradigms and theoretical underpinnings of scholarly work. While this may likely be due to space limitations associated with journal articles, clearly outlining philosophical foundations of research provide the reader with a frame through which to better understand the findings and implications of the research (Creswell, 2013). Clearly situating research in relation to theory is important for explaining and understanding an area of inquiry (Doherty, 2013). Moving forward, scholars should ensure their philosophical underpinnings and assumptions are clearly stated to better situate their work and demonstrate a clearer line of critical inquiry in the field.

### Sport Management Focus

#### Context

The studies included in this scoping review covered a wide range of contexts including: community sport (*n* = 39; 18%), sport events (*n* = 37; 17%), intercollegiate sport (*n* = 28; 13%), sport governance (*n* = 27; 13%), professional sport (*n* = 21; 10%), sport management academia (*n* = 21; 10%), sport for development (*n* = 20; 9%), sport media (*n* = 10; 5%), sport fans (*n* = 5; 2%), and other (*n* = 8; 4%). These results are not surprising given the varied accepted definitions for sport, and therefore sport management, depending on the personal beliefs and values of the authors, editors, and reviewers, and the mandates of specific journals. Further, the definition of sport varies depending upon which part of the world one is studying or referring to, as discussed in the introduction above (see DeSensi et al., 1990; Council of Europe, 2001; Coakley, 2003).

#### Area

Stemming from the definition of sport management applied in this study, the following four areas of sport management were identified: Education, Governance, Management, and Marketing. Based on these areas of sport management, additional analyses were run to further explore the use of critical research approaches in the field in a more nuanced manner. When limiting the categorization of the study context to the facets of the definition of sport management (i.e., education, governance, management, marketing), there is a clear emphasis on management (*n* = 89; 41%), followed by governance (*n* = 51; 24%), then marketing (*n* = 43; 20%), and education (*n* = 33; 15%) not far behind.

Of the 33 education articles, the majority (*n* = 18; 55%) appear in the sport management journals as opposed to journals of other domains. This is not surprising, as many of the education-centered articles are commenting on sport management as a field and education in sport management. Of the 51 governance articles, there are a similar number of articles in both the sport management (*n* = 19; 37%) and sport sociology (*n* = 15; 29%) domains. There is a fairly even distribution of articles in the other domains for the remainder of the governance articles. A similar trend is evident in the management (*n* = 89) area as well, as these articles were similarly published in sport management (*n* = 21; 24%) and sport sociology (*n* = 16; 18%) journals. For the management articles (*n* = 89), there are also several articles in the education (*n* = 10; 11%) and physical activity/leisure (*n* = 10; 11%) domains.

Interestingly, for the marketing domain (*n* = 43), there are more articles published in the sport sociology (*n* = 10; 23%) and communication (*n* = 9; 21%) domains than the sport management (*n* = 8; 19%) domain, and an equal number of articles in the psychology/sociology domain (*n* = 8; 19%). Marketing-driven articles in sociological oriented journals often rely on critical sociological theories such as Bourdieu's (1979) Theory of “La Distinction.” This indicates that perhaps marketing-driven articles published in the sport management domain tend to not take on a critical perspective. This may be because critical marketing scholars prefer to publish in non-sport management journals, or perhaps sport management journals prefer to publish non-critical marketing studies. Regardless, this finding suggests that there is room for growth and acceptance of critical marketing work in the sport management field, particularly targeting underserviced groups (i.e., target markets) in society.

### Methodological Approaches

#### Methods

In alignment with the critical paradigm, the largest proportion of studies included in the sample use a qualitative approach (*n* = 124; 57%). The next largest proportion of studies are conceptual pieces (*n* = 69; 32%), followed by mixed methods (*n* = 12; 6%), and then quantitative approaches (*n* = 7; 3%), and other (*n* = 4; 2%).

#### Study Design

The most popular study design among the studies included in the sample are conceptual papers (*n* = 69; 32%), indicating further potential for such conceptual pieces to be expanded upon empirically. Testing such conceptual pieces empirically could be a fruitful area of research to ensure the expansion of critical social science use in sport management moving forward. Of the empirical studies, the most frequently employed study designs are cross-sectional (*n* = 60; 28%), followed by case studies (*n* = 59; 27%), longitudinal (*n* = 14; 6%), ethnographic (*n* = 12; 6%), and finally, reviews (n=2; 1%).

#### Type of Data

There is a relative balance between conceptual pieces (*n* = 69; 32%), studies that use primary data (*n* = 64; 30%), secondary data (*n* = 62; 29%), and both (*n* = 21; 10%). This finding indicates the potential of employing critical social science for expanding how research is conducted in the field. This potential has been discussed previously (Frisby, 2005), and sport management scholars specifically have suggested that variety in research methods and approaches could further strengthen the field (Olafson, 1995; Sotiriadou and Shilbury, 2010; van der Roest et al., 2015; Sveinson et al., 2021). Therefore, this finding provides further reasoning for employing a critical approach in sport management work and the value of such approaches for the field.

#### Study Population

To accurately depict the distribution of populations within the collected articles, the percentages reported here reflect the total number of populations studied (*n* = 235) and not the total number of articles (*n* = 216) because some studies examined more than one population. The largest proportion are the non-empirical studies, without reference to specific populations (*n* = 69; 29%). In the empirical investigations, the largest proportion of studies considered administrators/organizations (*n* = 58; 25%), followed by media outlets and media documents (*n* = 38; 16%) that, combined, accounted for about two thirds of the empirical studies. Other populations consisted of amateur sport participants (adults and youth; *n* = 16; 8%), other types of documents (*n* = 18; 7%), elite athletes (*n* = 9; 4%), communities (*n* = 9; 4%), spectators (*n* = 6; 2%), and other (*n* = 12; 5%). This finding demonstrates that most critical research in the sport management field investigates the organizational level of sport. This clearly outlines a gap, and further suggests the need for future critical sport management research to involve diverse stakeholder groups (such as athletes, community members, BIPOC, etc.) to add to our holistic understanding of sport. For example, sport marketing and consumer behavior research could benefit from critical research approaches, particularly to better understand the power relations that prevent non-sport-participants from engaging in sport to inform how organizations could begin to remove barriers to participation.

#### Type of Analysis

Considering the empirical investigations included in this scoping review (*n* = 147), and the types of analyses conducted (*n* = 160; some investigations conducted more than one type of analysis) the most frequently used types of analysis were thematic (*n* = 50; 31%) followed by discourse/critical discourse (*n* = 35; 22%), and not stated (*n* = 34; 21%). Statistical (*n* = 11; 7%), content (*n* = 9; 6%), critical (*n* = 7; 4%), policy (*n* = 6; 4%), framing (*n* = 5; 3%), and other (*n* = 3; 2%) analyses were also used. Of note here is the finding that 21% of empirical articles collected for this scoping review did not state a type of analysis. This is noteworthy given the importance of demonstrating cohesiveness within projects to ensure quality of the research (Tracy, 2010). This finding indicates that researchers may need to be more explicit in terms of their critical research process and analysis to ensure the cohesiveness and quality of their work is demonstrated in manuscripts. By ensuring that quality criteria, particularly for qualitative research (Hoeber and Shaw, 2017; Hoeber et al., 2021), are met and explicitly outlined in research outputs may help to further demonstrate the value of diverse research approaches in the field.

### Outputs

#### Theoretical Implications

Most studies included in this scoping review (*n* = 180; 83%) did not specifically extend or develop the theory that was employed in the investigation. At best, only one third of the studies published in the administration/business domain developed theory (*n* = 3; 33%). Of the studies in the sport management domain, only 27% (*n* = 18) contributed to theory, followed by sport sociology (*n* = 6; 15%), geography/public health (*n* = 1; 13%), education (*n* = 2; 11%), psychology/sociology (*n* = 2; 10%), and communication (*n* = 1; 8%). None of the studies in physical activity/leisure contributed to theory development. While critical social science may have grown in popularity and acceptance within the sport management field since 1985, this finding suggests that there is further work to do in advancing such theories, extending their applicability to sport-specific contexts, and drawing upon the context of sport to extend theories for use in broader domains as well (Chalip, 2006). Theory improvement and extension, as well as theory development, are ways that sport management may contribute to broader literature in parent disciplines such as sociology, psychology, marketing, and administration (Gammelsæter, 2020).

## Conclusion

Over the past two decades, there have been calls from researchers in sport management to increase the use of critical social science and associated research approaches in the field. Frisby (2005) stated that using critical social science in sport management research would help to uncover the “ugly” side of sport, and from there, lasting change could be sought. Alongside such calls, scholars have begun to adopt more qualitative research methodologies, innovative theoretical approaches, and generally broaden research approaches in the field (e.g., Byers, 2013; Kitchin and Howe, 2013; Hoeber and Shaw, 2017; Chen and Mason, 2019). Despite these shifts, there had not yet been any systematic work investigating the state of the use of critical social science and associated research approaches in sport management scholarship. Employing critical social science in sport management research is important for unpacking the less-desirable aspects of sport as a social system and can therefore provide a strong base upon which positive change in sport can be made. As such, the purpose of our scoping review was to examine the trends, gaps, and the state of the use of critical social science and associated research approaches in sport management scholarship. By employing Arksey and O'Malley's (2005) scoping review methodology, and Teare and Taks (2020) systematic approach to scoping reviews, we collected and examined 216 unique articles.

Our findings suggest that while there has been an increase in critical social science in sport management since 1985 and particularly since 2005, there remains a relatively low number of critical social science published in sport management journals specifically per year. As such, there remains potential for scholars to adopt critical social science and associated methodologies in sport management scholarship, and for sport management journals to accept and publish such work, as discussed elsewhere (see Sveinson et al., 2021). Further, our findings suggest that while there is some critical work published, there is room for expanding the use of these approaches to include additional perspectives and sport contexts. Interestingly, our results demonstrate that there remains a need for scholars to intentionally state their paradigmatic and theoretical positions within research outputs. There currently seems to be a lack of outright communication regarding how critical social science is being used in sport management research. By ensuring research approaches are communicated clearly, readers will be better equipped to implement findings and to use existing work to push their own critical research forward. In doing so, more critical work will be readily available in sport management specific journals for sport management scholars, students, and practitioners to help inform future work and practice. Such critical academic work can support equity, diversity, and inclusion policy, the creation of safe sporting spaces, and ultimately support the provision of appropriate programming to as many diverse groups as possible.

### Future Research

This scoping review is a first step in better understanding trends, gaps, and the state of the use of critical research approaches in sport management scholarship. We acknowledge that given the nature of a scoping review there may be some articles that were not included as they did not explicitly state using a critical approach. Therefore, future work could employ a more targeted review approach by perhaps comparing publication trends in specific journals using full-text screening earlier in the search process. Specifically regarding the findings of this study, further research should uncover underlying reasons regarding author decisions to submit work to specific journals compared to others, as well as editor decisions to accept certain articles. Such work may uncover why critical sport management research is published more often in other journal domains as well as any constraints that may exist in publishing critical work in field-specific journals. Having a better understanding of the landscape of publication can help to create opportunities for increased critical scholarship in the field.

## Author Contributions

KB and GT were responsible for the conceptualization of the manuscript, data collection and analysis, and writing the manuscript. MT provided guidance on the conceptualization, data collection and analysis, writing, and also involved in adding to, editing and revising the final draft. All authors have made a substantial, direct, and intellectual contribution to the work and approved the submitted manuscript.

## Conflict of Interest

The authors declare that the research was conducted in the absence of any commercial or financial relationships that could be construed as a potential conflict of interest.

## Publisher's Note

All claims expressed in this article are solely those of the authors and do not necessarily represent those of their affiliated organizations, or those of the publisher, the editors and the reviewers. Any product that may be evaluated in this article, or claim that may be made by its manufacturer, is not guaranteed or endorsed by the publisher.

## Footnotes

^1^Note that ESMQ was preceded by the *European Journal for Sport Management*, which published it first issue in 1994. The *European Journal for Sport Management* is not available online and thus was not included in this scoping review.

## References

[B1] Alvesson M. Deetz S. (2000). Doing Critical Management Research, 1st Edn. Thousand Oaks, CA: Sage. 10.4135/9781849208918

[B2] Amis J. Silk M. (2005). Rupture: promoting critical and innovative approaches to the study of sport management. J. Sport Manag. 19, 355–366. 10.1123/jsm.19.4.355

[B3] Anderson E. McCormack M. (2010). Intersectionality, critical race theory, and american sporting oppression: examining black and gay male athletes. J. Homosexual. 57, 949–967. 10.1080/00918369.2010.503502 20818524

[B4] Arksey H. O'Malley L. (2005). Scoping studies: towards a methodological framework. Int. J. Soc. Res. Methodol. 8, 19–32. 10.1080/1364557032000119616

[B5] Bourdieu P. (1979). La distinction: Critique Sociale du Jugement. Paris: Minuit.

[B6] Buffington D. Fraley T. (2008). Skill in black and white: negotiating media images of race in a sporting context. J. Comm. Inq. 32, 292–310. 10.1177/0196859908316330

[B7] Byers T. (2013). Using critical realism: a new perspective on control of volunteers in sport clubs. Eur. Sport Manag. Q. 13, 5–31. 10.1080/16184742.2012.744765

[B8] Chalip L. (2006). Toward a distinctive sport management discipline. J. Sport Manag. 20, 1–21. 10.1123/jsm.20.1.1

[B9] Chen C. Mason D. S. (2019). Making settler colonialism visible in sport management. J. Sport Manag. 33, 379–392. 10.1123/jsm.2018-0243

[B10] Coakley J. (2003). Sport in Society: Issues and Controversies, 8th Edn. Boston, MA: McGraw Hill.

[B11] Cooper J. N. Grenier R. S. Macaulay C. (2017). Autoethnography as a critical approach in sport management: current applications and directions for future research. Sport Manag. Rev. 20, 43–54. 10.1016/j.smr.2016.07.003

[B12] Council of Europe (2001). European Sports Charter. Strasbourg: Council of Europe.

[B13] Creswell J. W. (2013). Research Design: Qualitative, Quantitative, and Mixed Methods Approaches, 4th Edn. Thousand Oaks, CA: Sage.

[B14] Cunningham G. Fink J. (2006). Diversity issues in sport and leisure. J. Sport Manag. 20, 455–465. 10.1123/jsm.20.4.455

[B15] DeSensi J. T. (1994). Multiculturalism as an issue in sport management. J. Sport Manag. 8, 63–74. 10.1123/jsm.8.1.63

[B16] DeSensi J. T. Kelley D. R. Blanton M. D. Beitel P. A. (1990). Sport management curricular evaluation and needs assessment: a multifaceted approach. J. Sport Manag. 4, 31–58. 10.1123/jsm.4.1.31

[B17] Doherty A. (2013). Investing in sport management: the value of good theory. Sport Manag. Rev. 16, 5–11. 10.1016/j.smr.2011.12.006

[B18] Dowling M. Leopkey B. Smith L. (2018). Governance in sport: a scoping review. J. Sport Manag. 32, 438–451. 10.1123/jsm.2018-0032

[B19] Downward P. (2005). Critical (realist) reflection on policy and management research in sport, tourism and sports tourism. Eur. Sport Manag. Q. 5, 303–320. 10.1080/16184740500190702

[B20] Engels T. C. E. Ossenblok T. L. B. Spruyt E. H. J. (2012). Changing publication patterns in the social sciences and humanities, 2000–2009. Scientometrics 93, 373–390. 10.1007/s11192-012-0680-2

[B21] Fink J. S. (2016). Hiding in plain sight: the embedded nature of sexism in sport. J. Sport Manag. 30, 1–7. 10.1123/jsm.2015-0278

[B22] Frisby W. (2005). The good, the bad, and the ugly: critical sport management research. J. Sport Manag. 19, 1–12. 10.1123/jsm.19.1.1

[B23] Frisby W. Reid C. J. Millar S. Hoeber L. (2005). Putting “participatory” into participatory forms of action research. J. Sport Manag. 19, 367–386. 10.1123/jsm.19.4.36712603626

[B24] Gammelsæter H. (2020). Sport is not industry: bringing sport back to sport management. Eur. Sport Manag. Q. 21, 1–23. 10.1080/16184742.2020.1741013

[B25] Gill E. L. Christensen M. C. Pérez A. G. (2017). The sale of the atlanta hawks: is it racism or white ownership playing the race card? J. Sports Med. 12, 113–140. 10.1353/jsm.2017.0005

[B26] Giulianotti R. (2015). Corporate social responsibility in sport: critical issues and future possibilities. Corp. Gov. 15, 243–248. 10.1108/CG-10-2014-0120

[B27] Grant M. J. Booth A. (2009). A typology of reviews: an analysis of 14 review types and associated methodologies: a typology of reviews. Health Info. Lib. J. 26, 91–108. 10.1111/j.1471-1842.2009.00848.x 19490148

[B28] Guba E. G. Lincoln Y. S. (1994). Competing Paradigms in Qualitative Research, in Handbook of Qualitative Research, eds Denzin N. K. Lincoln Y. S. (Thousand Oaks, CA: Sage), 105–117.

[B29] Hansell A. H. Giacobbi P. R. Voelker D. K. (2021). A scoping review of sport-based health promotion interventions with youth in Africa. Health Promo. Pract. 22, 31–40. 10.1177/1524839920914916 32264707 PMC7720243

[B30] Hoeber L. Kerwin S. (2013). Exploring the experiences of female sport fans: A collaborative self-ethnography. Sport Manag. Rev. 16, 326–336. 10.1016/j.smr.2012.12.002

[B31] Hoeber L. Shaw S. (2017). Contemporary qualitative research methods in sport management. Sport Manag. Rev. 20, 4–7. 10.1016/j.smr.2016.11.005

[B32] Hoeber L. Sveinson K. Shaw S. Misener K. Rich K. Chen C. (2021). Insights about publishing qualitative research from ‘reviewer 2’: conversations and collective learning, in 2021 North American Society for Sport Management Conference.

[B33] Inoue Y. Berg B. K. Chelladurai P. (2015). Spectator sport and population health: a scoping study. J. Sport Manag. 29, 705–725. 10.1123/JSM.2014-0283

[B34] Kincheloe J. L. McLaren P. (2011). Rethinking critical theory and qualitative research, in Key Works in Critical Pedagogy, eds Hayes K. Steinberg S. R. Tobin K. (Rotterdam, NL: Sense Publishers), 285–326. 10.1007/978-94-6091-397-6_23

[B35] Kitchin P. Howe D. (2013). How can the social theory of pierre bourdieu assist sport management research? Sport Manag. Rev. 16, 124–134. 10.1016/j.smr.2012.09.003

[B36] Knoppers A. (2015). Assessing the sociology of sport: on critical sport sociology and sport management. Int. Rev. Soc. Sport. 50, 496–501. 10.1177/1012690214538862

[B37] Lavelle K. L. (2010). A critical discourse analysis of black masculinity in NBA game commentary. Howard J. Comm. 21, 294–314. 10.1080/10646175.2010.496675

[B38] Levermore R. (2011). Sport-for-Development and the 2010 football world cup. Geog. Comp. 5, 886–897. 10.1111/j.1749-8198.2011.00460.x

[B39] Levermore R. Moore N. (2015). The need to apply new theories to “sport CSR”. Corp. Gov. 15, 249–253. 10.1108/CG-09-2014-0113

[B40] Ličen S. Billings A. C. (2013). Cheering for ‘our’ champs by watching ‘sexy’ female throwers: representation of nationality and gender in Slovenian 2008 summer olympic television coverage. Eur. J. Comm. 28, 379–396. 10.1177/0267323113484438

[B41] Masucci M. Butryn T. M. (2013). Writing about fighting: a critical content analysis of newspaper coverage of the ultimate fighting championship from 1993-2006. J. Sports Media. 8, 19–44. 10.1353/jsm.2013.0005

[B42] McGarry J. E. (2020). Enact, discard, transform: an impact Agenda. J. Sport Manag. 34, 1–8. 10.1123/jsm.2019-0391

[B43] McGillivray D. McPherson G. Misener L. (2018). Major sporting events and geographies of disability. Urb. Geog. 39, 329–344. 10.1080/02723638.2017.1328577

[B44] McSweeney M. Faust K. (2019). How do you know if you don't try*?* Non-traditional research methodologies, novice researchers, and leisure studies. Leisure 43, 339–364. 10.1080/14927713.2019.162983031182117

[B45] North American Society for Sport Management (n.d.). Purpose & History. Available online at: https://www.nassm.com/NASSM/Purpose (accessed July 30, 2020).

[B46] Olafson G. A. (1995). Sport management research: ordered change. J. Sport Manag. 9, 338–345. 10.1123/jsm.9.3.338

[B47] Prouse C. (2015). Harnessing the hijab: the emergence of the muslim female footballer through international sport governance. Gender Place Cult. J. Fem. Geog. 22, 20–36. 10.1080/0966369X.2013.832664

[B48] Quatman C. Chelladurai P. (2008). Social network theory and analysis: a complementary lens for inquiry. J. Sport Manag. 22, 338–360. 10.1123/jsm.22.3.338

[B49] Rankin-Wright A. J. Hylton K. Norman L. (2016). Off-colour landscape: Framing race equality in sport coaching. Soc. Sport J. 33, 357–368. 10.1123/ssj.2015-0174

[B50] Samatas M. (2011). Surveillance in Athens 2004 and Beijing 2008: a comparison of the olympic surveillance modalities and legacies in two different olympic host regimes. Urb. Stud. 48, 3347–3366. 10.1177/0042098011422399

[B51] Shaw S. (2019). The chaos of inclusion? Examining anti-homophobia policy development in New Zealand sport. Sport Manag. Rev. 22, 247–262. 10.1016/j.smr.2018.04.001

[B52] Shaw S. Frisby W. (2006). Can gender equity be more equitable?: promoting an alternative frame for sport management research, education, and practice. J. Sport Manag. 20, 483–509. 10.1123/jsm.20.4.483

[B53] Shaw S. Hoeber L. (2016). Unclipping our wings: ways forward in qualitative research in sport management. Sport Manag. Rev. 19, 255–265. 10.1016/j.smr.2016.03.001

[B54] Singer J. N. (2005). Addressing epistemological racism in sport management research. J. Sport Manag. 19, 464–479. 10.1123/jsm.19.4.464

[B55] Singer J. N. Shaw S. Hoeber L. Walker N. Agyemang K. J. A. Rich K. (2019). Critical conversations about qualitative research in sport management. J. Sport Manag. 33, 50–63. 10.1123/jsm.2018-008527939622

[B56] Skinner J. Edwards A. (2005). Inventive pathways: fresh visions of sport management research. J. Sport Manag. 19, 404–421. 10.1123/jsm.19.4.404

[B57] Sotiriadou K. Shilbury D. (2010). Using grounded theory in sport management research. Int. J. Sport Manag. Mark. 8, 181–202. 10.1504/IJSMM.2010.03750335009967

[B58] Sveinson K. Hoeber L. Heffernan C. (2021). Critical discourse analysis as theory, methodology, and analyses in sport management studies. J. Sport Manag. 35, 465–475. 10.1123/jsm.2020-0288

[B59] Teare G. Taks M. (2020). Extending the scoping review framework: a guide for interdisciplinary researchers. Int. J. Soc. Res. Methodol. 23, 311–315. 10.1080/13645579.2019.1696092

[B60] Toffoletti K. (2017). Sexy women sports fans: femininity, sexuality, and the global sport spectacle. Fem. Media Stud. 17, 457–472. 10.1080/14680777.2016.1234499

[B61] Tracy S. J. (2010). Qualitative quality: Eight “big-tent” criteria for excellent qualitative research. Qual. Inq. 16, 837–851. 10.1177/1077800410383121

[B62] Travers A. (2011). Women's ski jumping, the 2010 olympic games, and the deafening silence of sex segregation, whiteness, and wealth. J. Sport Soc. Iss. 35, 126–145. 10.1177/0193723511405477

[B63] van der Roest J.-W. Spaaij R. van Bottenburg M. (2015). Mixed methods in emerging academic subdisciplines: the case of sport management. J. Mix. Meth. Res. 9, 70–90. 10.1177/1558689813508225

[B64] Warner S. (2019). Sport as medicine: how F3 is building healthier men and communities. Sport Manag. Rev. 22, 38–52. 10.1016/j.smr.2018.06.006

[B65] Whittemore R. Chao A. Jang M. Minges K. E. Park C. (2014). Methods for knowledge synthesis: an overview. Heart Lung. 43, 453–461. 10.1016/j.hrtlng.2014.05.014 25012634

[B66] Zipp S. Smith T. Darnell S. (2019). Development, gender and sport: theorizing a feminist practice of the capabilities approach in sport for development. J. Sport Manag. 33, 440–449. 10.1123/jsm.2019-0126

