# Supplementary material for: Critical Social Science in Sport Management Research: A Scoping Review
Source: Front Sports Act Living. 2022 Jan 28;4:812200. doi: 10.3389/fspor.2022.812200 (PMC8831802; doi:10.3389/fspor.2022.812200)
Supplement: Supplementary file 1 [file Table_1.DOCX]

***Supplementary Material***

Supplementary Table 1: Critical Social Science Publications in Sport Management Included in the Scoping Review

| **Search** | **Article** |
| --- | --- |
| Both | Agergaard, S., Michelsen la Cour, A., & Gregersen, M. T. (2016). Politicisation of migrant leisure: A public and civil intervention involving organised sports. *Leisure Studies, 35*(2), 200–214. |
| Both | Agyemang, K., Singer, J. N., & DeLorme, J. (2010). An exploratory study of black male college athletes’ perceptions on race and athlete activism. *International Review for the Sociology of Sport, 45*(4), 419–435. |
| Both | Aitchison, C. C. (2005). Feminist and gender research in sport and leisure management: Understanding the social–cultural nexus of gender–power relations. *Journal of Sport Management, 19*(4), 422–441. |
| Both | Amis, J., & Silk, M. L. (2005). Rupture: Promoting critical and innovative approaches to the study of sport management. *Journal of Sport Management, 19*(4), 355–366. |
| Both | Apostolis, N., & Giles, A. R. (2011). Portrayals of women golfers in the 2008 issues of golf digest. *Sociology of Sport Journal, 28*(2), 226–238. |
| Both | Bain, L. L. (1989). Interpretive and critical research in sport and physical education. *Research Quarterly for Exercise and Sport, 60*(1), 21–24. |
| Both | Bimper, A. Y., & Harrison, L. (2017). Are we committed to issues of race? Institutional integrity across intercollegiate athletics*. International Review for the Sociology of Sport, 52*(6), 675–692. |
| Both | Boshaff, G. B. E. (1997). "Barefoot” sports administrators: Laying the foundation for sports development in South Africa. *Journal of Sport Management, 11*(1), 69–79. |
| Both | Boucher, L. (2015). Public emotions and their personal consequences: The nationalizing affects of the Australian Football League since 1990. *The International Journal of the History of Sport, 32*(13), 1546–1566. |
| Both | Boykoff, J., & Yasuoka, M. (2015). Gender and politics at the 2012 Olympics: Media coverage and its implications. *Sport in Society, 18*(2), 219–233. |
| Both | Brown, K., & Williams, A. (2018). Out of bounds: A critical race theory perspective on ‘pay for play.’ *Journal of Legal Aspects of Sport, 29*(1). |
| Both | Burdsey, D. (2014). One week in October: Luis Suárez, John Terry and the turn to racial neoliberalism in English men’s professional football. *Identities, 21*(5), 429–447. |
| Both | Carey, R. S. (2013). Hoosier Whiteness and the Indiana Pacers: Racialized strategic change and the politics of organizational sensemaking. *Sport in Society, 16*(5), 631–653. |
| Both | Chalip, L. (1996). Critical Policy Analysis: The illustrative case of New Zealand sport policy development. *Journal of Sport Management, 10*(3), 310–324. |
| Both | Cooper, J. N., Grenier, R. S., & Macaulay, C. (2017). Autoethnography as a critical approach in sport management: Current applications and directions for future research. *Sport Management Review, 20*(1), 43–54. |
| Both | Cooper, J. N., Nwadike, A., & Macaulay, C. (2017). A critical race theory analysis of big-time college sports: Implications for culturally responsive and race-conscious sport leadership. *Journal of Issues in Intercollegiate Athletics, 10*, 204–233. |
| Both | Dowling, M. (2018). Exploring Sport management as an academic profession: A critical review of occupational theory. *Journal of Global Sport Management, 3*(4), 321–338. |
| Both | Frisby, W. (2005). The good, the bad, and the ugly: Critical sport management research. *Journal of Sport Management, 19*(1), 1–12. |
| Both | Gee, S. (2013). The culture of alcohol sponsorship during the 2011 Rugby World Cup: An (auto)ethnographic and (con)textual analysis. *Sport in Society, 16*(7), 912–930. |
| Both | Gee, S. (2014). Bending the codes of masculinity: David Beckham and flexible masculinity in the new millennium. *Sport in Society, 17*(7), 917–936. |
| Both | Georgoulas, S. (2013). Social control in sports and the CCTV issue: A critical criminological approach. *Sport in Society, 16*(2), 239–249. |
| Both | Giulianotti, R., & Klauser, F. (2010). Security governance and sport mega-events: Toward an interdisciplinary research agenda. *Journal of Sport and Social Issues, 34*(1), 49–61. |
| Both | Giulianotti, R., & Klauser, F. (2012). Sport mega-events and ‘terrorism’: A critical analysis. *International Review for the Sociology of Sport, 47*(3), 307–323. |
| Both | Glover, T. D. (2007). Ugly on the diamonds: An examination of white privilege in youth baseball. *Leisure Sciences, 29*(2), 195–208. |
| Both | Griffin, R. A. (2012). The disgrace of commodification and shameful convenience: A critical race critique of the NBA. *Journal of Black Studies, 43*(2), 161–185. |
| Both | Guan, Z. (2015). Paralympics in China: A social approach versus an elite approach. *The International Journal of the History of Sport, 32*(8), 1115–1120. |
| Both | Hall, M. A., Cullen, D., & Slack, T. (1989). Organizational elites recreating themselves: The gender structure of national sport organizations. *Quest, 41*(1), 28–45. |
| Both | Hayhurst, L. M. C., & Szto, C. (2016). Corporatizating activism through sport-focused social justice? Investigating Nike’s corporate responsibility initiatives in sport for development and peace. *Journal of Sport and Social Issues, 40*(6), 522–544. |
| Both | Hylton, K., & Morpeth, N. D. (2012). London 2012: ‘Race’ matters and the East End. *International Journal of Sport Policy and Politics, 4*(3), 379–396. |
| Both | Kane, M. J., & Maxwell, H. D. (2011). Expanding the boundaries of sport media research: Using critical theory to explore consumer responses to representations of women’s sports*. Journal of Sport Management, 25*(3), 202–216. |
| Both | King, A. (1997). New directors, customers, and fans: The transformation of English football in the 1990s. *Sociology of Sport Journal, 14*(3), 224–240. |
| Both | Kitchin, P. J., & David Howe, P. (2013). How can the social theory of Pierre Bourdieu assist sport management research? *Sport Management Review, 16*(2), 123–134. |
| Both | Knoppers, A. (2015). Assessing the sociology of sport: On critical sport sociology and sport management. *International Review for the Sociology of Sport, 50*(4–5), 496–501. |
| Both | Law, A. (2014). Playing with tension: National charisma and disgrace at Euro 2012. *Soccer & Society, 15*(2), 203–221. |
| Both | Lawrence, S., & Davis, C. (2019). Fans for diversity? A Critical Race Theory analysis of Black, Asian and Minority Ethnic (BAME) supporters’ experiences of football fandom. International *Journal of Sport Policy and Politics, 11*(4), 701–713. |
| Both | Levermore, R., & Moore, N. (2015). The need to apply new theories to “Sport CSR.” *Corporate Governance;* *Bradford, 15*(2), 249–253. |
| Both | Long, J., Robinson, P., & Spracklen, K. (2005). Promoting racial equality within sports organizations. *Journal of Sport and Social Issues, 29*(1), 41–59. |
| Both | Lusted, J. (2018). A critical realist morphogenetic approach to researching sport policy: Reflections on a large-scale study of policy implementation in grassroots English football. *International Journal of Sport Policy and Politics, 10*(4), 705–719. |
| Both | Lynch, M., & Yerashotis, G. (2019). Sporting chancers: Three Canadian corporations’ representations of sport-for-youth-development. *International Review for the Sociology of Sport, 54*(6), 666–690. |
| Both | MacDonald, M. N., & Hunter, D. (2013). The discourse of Olympic security: London 2012. *Discourse & Society, 24*(1), 66–88. |
| Both | McPherson, G., O’Donnell, H., McGillivray, D., & Misener, L. (2016). Elite athletes or superstars? Media representation of para-athletes at the Glasgow 2014 Commonwealth Games. *Disability & Society, 31*(5), 659–675. |
| Both | Meân, L. J., Kassing, J. W., & Sanderson, J. (2010). The making of an epic (American) hero fighting for justice: Commodification, consumption, and intertextuality in the Floyd Landis defense campaign. *American Behavioral Scientist, 53*(11), 1590–1609. |
| Both | Misener, L. (2015). Leveraging parasport events for community participation: Development of a theoretical framework. *European Sport Management Quarterly, 15*(1), 132–153. |
| Both | Njelesani, J., Cameron, D., Gibson, B. E., Nixon, S., & Polatajko, H. (2014). A critical occupational approach: Offering insights on the sport-for-development playing field. *Sport in Society*. |
| Both | Pope, S. W. (2010). Embracing cultural contexts and critical reflexivity: (Re)presenting the global sports industry in research and practice. *European Sport Management Quarterly, 10*(4), 509–524. |
| Both | Prouse, C. (2015). Harnessing the hijab: The emergence of the Muslim Female Footballer through international sport governance. *Gender, Place & Culture, 22*(1), 20–36. |
| Both | Rich, K. A., & Giles, A. R. (2015). Managing diversity to provide culturally safe sport programming: A case study of the Canadian Red Cross’s swim program. *Journal of Sport Management, 29*(3), 305–317. |
| Both | Rich, K.A, & Giles, A. R. (2014). Examining whiteness and Eurocanadian discourses in the Canadian Red Cross’ swim program. *Journal of Sport and Social Issues, 38*(5), 465–485. |
| Both | Shaw, S. (2009). “It was all ‘smile for Dunedin!”’: Event volunteer experiences at the 2006 New Zealand Masters Games. *Sport Management Review, 12*(1), 26–33. |
| Both | Shaw, S. (2019). The chaos of inclusion? Examining anti-homophobia policy development in New Zealand sport. *Sport Management Review, 22*(2), 247–262. |
| Both | Singer, J. N. (2005). Addressing epistemological racism in sport management research. *Journal of Sport Management, 19*(4), 464. |
| Both | Singer, J. N. (2009). African American football athletes’ perspectives on institutional integrity in college sport. *Research Quarterly for Exercise and Sport, 80*(1), 102–116. |
| Both | Singer, J. N., Harrison, C. K., & Bukstein, S. J. (2010). A Critical Race analysis of the hiring process for head coaches in NCAA college football. *Journal of Intercollegiate Sport, 3*(2), 270–296. |
| Both | Skinner, J., & Edwards, A. (2005). Inventive pathways: Fresh visions of sport management research. *Journal of Sport Management, 19*(4), 404. |
| Both | Spaaij, R., & Jeanes, R. (2013). Education for social change? A Freirean critique of sport for development and peace. *Physical Education & Sport Pedagogy, 18*(4), 442–457. |
| Both | Spaaij, R., Oxford, S., & Jeanes, R. (2016). Transforming communities through sport? Critical pedagogy and sport for development. *Sport, Education and Society, 21*(4), 570–587. |
| Both | Staurowsky, E. J. (1998). An act of honor or exploitation? The Cleveland Indians’ use of the Louis Francis Sockalexis story. *Sociology of Sport Journal, 15*(4), 299–316. |
| Both | Travers, A. (2011). Women’s ski jumping, the 2010 Olympic Games, and the deafening silence of sex segregation, whiteness, and wealth. *Journal of Sport and Social Issues, 35*(2), 126–145. |
| Both | Van Rheenen, D. (2014). A skunk at the garden party: The Sochi Olympics, state-sponsored homophobia and prospects for human rights through mega sporting events. *Journal of Sport & Tourism, 19*(2), 127–144. |
| Both | Villalon, C., & Weiller-Abels, K. (2018). NBC’s televised media portrayal of female athletes in the 2016 Rio Summer Olympic Games: A critical feminist view. *Sport in Society, 21*(8), 1137–1157. |
| Both | Warner, S. (2019). Sport as medicine: How F3 is building healthier men and communities. *Sport Management Review, 22*(1), 38–52. |
| Both | Zervas, K., & Glazzard, J. (2018). Sport management student as producer: Embedding critical management studies in sport through contemporary pedagogy. *Sport, Education and Society, 23*(9), 928–937. |
| Database | Açıkgöz, S., Haudenhuyse, R., & Aşçı, H. (2019). Social inclusion for whom and towards what end? A critical discourse analysis of youth and sport policies in Turkey. *Journal of Youth Studies, 22*(3), 330–345. |
| Database | Adams, A., & Harris, K. (2014). Making sense of the lack of evidence discourse, power and knowledge in the field of sport for development. *The International Journal of Public Sector Management; Bradford, 27*(2), 140–151. |
| Database | Adams, C., & Stevens, J. (2007). Change and grassroots movement: Reconceptualising women’s hockey governance in Canada. *International Journal of Sport Management and Marketing, 2*(4), 344. |
| Database | Aggestal, A., & Fahlen, J. (2015). Managing sport for public health: Approaching contemporary problems with traditional solutions. *Social Inclusion.* |
| Database | Arsenault, D. J., & Fawzy, T. (2001). Just Buy It: Nike advertising aimed at glamour readers: A Critical Feminist Analysis. *Tamara: Journal for Critical Organization Inquiry, 1*(2), Article 2. |
| Database | Baker-Lewton, A., Sonn, C. C., Vincent, D. N., & Curnow, F. (2017). ‘I haven’t lost hope of reaching out … ’: Exposing racism in sport by elevating counternarratives. *International Journal of Inclusive Education, 21*(11), 1097–1112. |
| Database | Bercovitz, K. L. (2000). A critical analysis of Canada’s ‘Active Living’: Science or politics? *Critical Public Health, 10*(1), 19–39. |
| Database | Berg, B. K., Warner, S., & Das, B. M. (2015). What about sport? A public health perspective on leisure-time physical activity. *Sport Management Review, 18*(1), 20–31. |
| Database | Bradbury, S., van Sterkenburg, J., & Mignon, P. (2018). The under-representation and experiences of elite level minority coaches in professional football in England, France and the Netherlands. *International Review for the Sociology of Sport, 53*(3), 313–334. |
| Database | Brennen, B., & Brown, R. (2016). Persecuting Alex Rodriguez: Race, money and the ethics of reporting the performance-enhancing drug scandal. *Journalism Studies, 17*(1), 21–38. |
| Database | Burdsey, D. (2016). One guy named Mo: Race, nation and the London 2012 Olympic Games. *Sociology of Sport Journal, 33*(1), 14–25. |
| Database | Burnett, C. (2014.). The intersecting life worlds of Sport for development (SfD) coaches in the Kiambiu slum of Nairobi, Kenya. *African Journal for Physical Health Education, Recreation and Dance, 20*(3), 963-973. |
| Database | Castañeda, L., & Sherrill, C. (1999). Family participation in Challenger Baseball: Critical theory perspectives. *Adapted Physical Activity Quarterly, 16*(4), 372–388. |
| Database | Cheeks, G., & Carter-Francique, A. R. (2015). HBCUS versus HWCUS: A critical examination of institutional distancing between collegiate athletic programs. *Race, Gender & Class, 22*(1/2), 23–35. |
| Database | Darnell, S. C., & Millington, R. (2019). Social justice, sport, and sociology: A position statement. *Quest, 71*(2), 175–187. |
| Database | Davis, L. R. (1993). Critical analysis of the popular media and the concept of ideal subject position: Sports Illustrated as case study. *Quest, 45*(2), 165–181. |
| Database | Edwards, A. (1999). Reflective practice in sport management. *Sport Management Review, 2*(1), 67–81. |
| Database | Edwards, A., Skinner, J., & Gilbert, K. (2005). Towards a critical theory of sport management. *International Journal of Sport Management, 6*(3), 233 |
| Database | Falcous, M. (2015). White is the new black? Football, media and the New Zealand imagination. *Soccer & Society, 16*(4), 555–572. |
| Database | Fay, T. (2011). Disability in sport it’s our time: From the sidelines to the frontlines (Title IX—B). *Journal of Intercollegiate Sport, 4*(1), 63–94. |
| Database | Giulianotti, R. (2015). Corporate social responsibility in sport: Critical issues and future possibilities. *Corporate Governance, 15*(2), 243–248. |
| Database | Giulianotti, R., Collison, H., Darnell, S., & Howe, D. (2017). Contested states and the politics of sport: The case of Kosovo – division, development, and recognition. *International Journal of Sport Policy and Politics, 9*(1), 121–136. |
| Database | Gong, Y. (2016). Online discourse of masculinities in transnational football fandom: Chinese Arsenal fans’ talk around ‘gaofushuai’ and ‘diaosi.’ *Discourse & Society, 27*(1), 20–37. |
| Database | Harvey, J., & Saint-Germain, M. (2001). Sporting goods trade, international division of labor, and the unequal hierarchy of nations. *Sociology of Sport Journal, 18*(2), 231–246. |
| Database | Hayhurst, L. M. C. (2009). The power to shape policy: Charting sport for development and peace policy discourses. *International Journal of Sport Policy and Politics, 1*(2), 203–227. |
| Database | Jacobs, J. M., Wright, P. M., Ressler, J. D., & Jung, J. (2016). Coaches’ learning outcomes in a Belizean sport for development program. *Research Quarterly for Exercise and Sport, 87*(S2), A95–A96. |
| Database | Kane, M. J., & Maxwell, H. D. (2011). Expanding the boundaries of sport media research: Using Critical Theory to explore consumer responses to representations of women’s sports*. Journal of Sport Management, 25*(3), 202–216. |
| Database | Lavelle, K. L. (2010). A critical discourse analysis of black masculinity in NBA game commentary. *Howard Journal of Communications, 21*(3), 294–314. |
| Database | Lawrence, S. (2016). ‘We are the boys from the Black Country’! (Re)Imagining local, regional and spectator identities through fandom at Walsall Football Club. *Social & Cultural Geography, 17*(2), 282–299. |
| Database | Ličen, S., & Billings, A. C. (2013). Cheering for ‘our’ champs by watching ‘sexy’ female throwers: Representation of nationality and gender in Slovenian 2008 Summer Olympic television coverage. *European Journal of Communication, 28*(4), 379–396. |
| Database | Lin, Y. (2013). A Critical Review of Social Impacts of Mega-events. *International Journal of Sport & Society, 3*(3). |
| Database | McDonald, I. (2005). Theorising partnerships: Governance, communicative action and sport policy. *Journal of Social Policy, 34*, 579–600. |
| Database | McLean, R., & Wainwright, D. W. (2009). Social networks, football fans, fantasy and reality: How corporate and media interests are invading our lifeworld. *Journal of Information, Communication & Ethics in Society, 7*(1), 54–71. |
| Database | Mwaanga, O., & Prince, S. (2016). Negotiating a liberative pedagogy in sport development and peace: Understanding consciousness raising through the Go Sisters programme in Zambia. *Sport, Education and Society, 21*(4), 588–604. |
| Database | Nam, B. H., Hong, D., Marshall, R. C., & Hong, J. (2018). Conflicts among stakeholders regarding the new academic system in the Korea University Sport Federation*. International Journal of Sport Policy and Politics, 10*(3), 597–613. |
| Database | Njelesani, J., Gibson, B. E., Cameron, D., Nixon, S., & Polatajko, H. (2015). Sport-for-Development: A Level Playing Field? *Qualitative Social Research, 16*(2), Article 2. |
| Database | Northcote, J., & Casimiro, S. (2009). A critical approach to evidence-based resettlement policy: Lessons learned from an Australian Muslim refugee sports program. *Tamara Journal of Critical Organisation Inquiry, 8*(1/2), 173–185. |
| Database | Piggin, J., Tlili, H., & Louzada, B. H. (2017). How does health policy affect practice at a sport mega event? A study of policy, food and drink at Euro 2016. *International Journal of Sport Policy and Politics, 9*(4), 739–751. |
| Database | Rankin-Wright, A. J., Hylton, K., & Norman, L. (2016). Off-colour landscape: Framing race equality in sport coaching. *Sociology of Sport Journal, 33*(4), 357–368. |
| Database | Rasul, A., & Proffitt, J. M. (2011). Bollywood and the Indian Premier League (IPL): The political economy of Bollywood’s new blockbuster. *Asian Journal of Communication, 21*(4), 373–388. |
| Database | Roper, E. A. (2009). On the sidelines: Roles and responsibilities of the Diamond Dolls in intercollegiate baseball. *Women in Sport & Physical Activity Journal, 18*(2), 52-. |
| Database | Shaw, A. A., Moiseichik, M., Blunt-Vinti, H., & Stokowski, S. (2019). Measuring racial competence in athletic academic support staff members. *Sociology of Sport Journal, 36*(2), 162–170. |
| Database | Shaw, S., Wolfe, R., & Frisby, W. (2011). A critical management studies approach to sport management education: Insights, challenges and opportunities. *Sport Management Education Journal, 5*(1), 1–13. |
| Database | Sabirova, G., & Zinoviev, A. (2016). Urban local sport clubs, migrant children and youth in Russia. *Community Development Journal*, *51*(4), 482-498. |
| Database | Skinner, J., Stewart, B., & Edwards, A. (1999). Amateurism to professionalism: Modelling organisational change in sporting organisations. *Sport Management Review, 2*(2), 173–192. |
| Database | Tomlinson, A. (2014). Olympic legacies: Recurrent rhetoric and harsh realities. *Contemporary Social Science, 9*(2), 137–158. |
| Database | Tynan, M., & Briggs, P. (2013). How culturally competent is the Australian Football League (AFL)? *International Journal of Sport & Society, 3*(3), 191–205. |
| Database | Lin, Y. (2013). A Critical Review of Social Impacts of Mega-events. *International Journal of Sport & Society, 3*(3). |
| Database | Westkott, M., & Coakley, J. J. (1981). Women in sport: modalities of feminist social change. *Journal of Sport and Social Issues, 5*(1), 32–45. |
| Database | Yassim, M. (2013). Cricket as a vehicle for community cohesion: Building bridges with British Muslims. *Journal of Islamic Marketing; Bingley, 4*(2), 218–227. |
| Manual | Adams, A., Anderson, E., & McCormack, M. (2010). Establishing and challenging masculinity: The influence of gendered discourses in organized sport. *Journal of Language and Social Psychology, 29*(3), 278–300. |
| Manual | Adriaanse, J. A. (2019). The influence of gendered emotional relations on gender equality in sport governance. *Journal of Sociology, 55*(3), 587–603. |
| Manual | Agyemang, K., & DeLorme, J. (2010). Examining the dearth of black head coaches at the NCAA football bowl subdivision level: A Critical Race Theory and Social Dominance Theory analysis. *Journal of Issues in Intercollegiate Athletics.* |
| Manual | Amara, M., & Henry, I. P. (2010). Sport, Muslim identities and cultures in the UK, an emerging policy issue: Case studies of Leicester and Birmingham*. European Sport Management Quarterly, 10*(4), 419–443. |
| Manual | Andrews, D. L., & Mower, R. L. (2012). Spectres of Jordan. *Ethnic and Racial Studies, 35*(6), 1059–1077. |
| Manual | Armstrong, K. L. (2007). The nature of Black women’s leadership in community recreation sport: An illustration of Black feminist thought. *Women in Sport & Physical Activity Journal, 16*(1), 3-. |
| Manual | Baker, A. C., & Giles, A. R. (2008). Pedagogy of the front float: Dialogue and aquatics programming in Taloyoak, Nunavut. *Arctic*. |
| Manual | Bar-On, T., & Escobedo, L. (2019). FIFA seen from a postcolonial perspective. *Soccer & Society, 20*(1), 39–60. |
| Manual | Berg, B. K., & Chalip, L. (2013). Regulating the emerging: A policy discourse analysis of mixed martial arts legislation. *International Journal of Sport Policy and Politics, 5*(1), 21–38. |
| Manual | Betzer-Tayar, M., Zach, S., Galily, Y., & Henry, I. (2017). Barriers to women’s access to decision-making positions in sport organizations: The case of establishing a girls’ volleyball academy in Israel. *Journal of Gender Studies, 26*(4), 418–431. |
| Manual | Bradbury, S. (2013). Institutional racism, whiteness and the under-representation of minorities in leadership positions in football in Europe. *Soccer & Society, 14*(3), 296–314. |
| Manual | Buffington, D., & Fraley, T. (2008). Skill in black and white: Negotiating media images of race in a sporting context. *Journal of Communication Inquiry, 32*(3), 292–310. |
| Manual | Burton, L. J., Borland, J., & Mazerolle, S. M. (2012). “They cannot seem to get past the gender issue”: Experiences of young female athletic trainers in NCAA Division I intercollegiate athletics. *Sport Management Review, 15*(3), 304–317. |
| Manual | Byers, T. (2013). Using critical realism: A new perspective on control of volunteers in sport clubs. *European Sport Management Quarterly, 13*(1), 5–31. |
| Manual | Carter-Francique, A. R., Dortch, D., & Carter-Phiri, K. (2017). Black female college athletes’ perception of power in sport and society. *Journal for the Study of Sports and Athletes in Education, 11*(1), 18–45. |
| Manual | Chen, C., & Mason, D. S. (2018). A Postcolonial reading of representations of non-western leadership in sport management studies. *Journal of Sport Management, 32*(2), 150–169. |
| Manual | Chen, C., & Mason, D. S. (2019). Making settler colonialism visible in sport management. *Journal of Sport Management, 33*, 379–392. |
| Manual | Chen, C., Mason, D. S., & Misener, L. (2018). Exploring media coverage of the 2017 World Indigenous Nations Games and North American Indigenous Games: A critical discourse analysis. *Event Management, 22,* 1009–1025. |
| Manual | Cooper, J. N., & Cooper, J. E. (2015). Success in the shadows: (Counter) narratives of achievement from black scholar athletes at a historically black College/University. *Journal for the Study of Sports and Athletes in Education, 9*(3), 145–171. |
| Manual | Darnell, S. C., & Hayhurst, L. M. C. (2011). Sport for decolonization: Exploring a new praxis of sport for development*. Progress in Development Studies, 11*(3), 183–196. |
| Manual | Devine, C. (2018). Sex, sport and money: Voice, choice and distributive justice in England, Scotland and Wales. *Sport, Education and Society, 23*(9), 824–839. |
| Manual | Downs, B. J., & Love, A. (2017). Everyone bleeds maroon: Colorblindness and the desegregation of Mississippi State Football. *International Journal of Sport Communication, 10*(4), 444–467. |
| Manual | Downward, P. (2005). Critical (realist) reflection on policy and management research in sport, tourism and sports tourism. *European Sport Management Quarterly, 5*(3), 303–320. |
| Manual | Engh, M. H., & Potgieter, C. (2018). Hetero-sexing the athlete: Public and popular discourses on sexuality and women’s sport in South Africa. *Acta Academica, 50*(2), 34–51. |
| Manual | Falcous, M., & Maguire, J. (2006). Imagining ‘America’: The NBA and local–global mediascapes. *International Review for the Sociology of Sport, 41*(1), 59–78. |
| Manual | Ferrand, C., Henry, I., & Ferrand, A. (2010). Gendered identities in self-descriptions of electoral candidates in a French National Sport Federation. *European Sport Management Quarterly*, *10*(5), 531-552. |
| Manual | Ferrand, C., Henry, I., & Ferrand, A. (2010). Gendered identities in self-descriptions of electoral candidates in a French National Sport Federation. *European Sport Management Quarterly, 10*(5), 531–552. |
| Manual | Finlay, C. J. (2018). The right to profitable speech: Olympians, sponsorship, and social media discourse. *Communication & Sport, 6*(6), 655–679. |
| Manual | Frisby, W., Crawford, S., & Dorer, T. (1997). Reflections on participatory action research: The case of low-income women accessing local physical activity services*. Journal of Sport Management, 11*(1), 8–28. |
| Manual | Frisby, W., Reid, C. J., Millar, S., & Hoeber, L. (2005). Putting “Participatory” into Participatory Forms of Action Research. *Journal of Sport Management, 19*(4), 367–386. |
| Manual | Gill, E. L., Christensen, M. C., & Pérez, A. G. (2017). The sale of the Atlanta Hawks: Is it racism or white ownership playing the racecCard? *Journal of Sports Media, 12*(1), 113–140. |
| Manual | Gill, Jr., Emmett L. (2011). The Rutgers Women’s Basketball & Don Imus Controversy (RUIMUS): White privilege, new racism, and the implications for college sport management. *Journal of Sport Management, 25*(2), 118–130. |
| Manual | Goodwin, M., & Grix, J. (2011). Bringing structures back in: The ‘governance narrative’, the ‘decentred approach’ and ‘asymmetrical network governance’ in the education and sport policy communities. *Public Administration, 89*(2), 537–556. |
| Manual | Grace, A. N., & Mueller, T. S. (2019). Gender bias in sport media: A critical analysis of Twitter content and the National Football League’s Carolina Panthers. *Journal of Gender Studies, 28*(3), 363–370. |
| Manual | Green, M. (2006). From ‘sport for all’ to not about ‘sport’ at all?: Interrogating sport policy interventions in the United Kingdom. *European Sport Management Quarterly, 6*(3), 217–238. |
| Manual | Halone, K. K. (2008). The structuration of racialized sport organizing*. Journal of Communication Inquiry, 32*(1), 22–42. |
| Manual | Harris, J., & Clayton, B. (2008). Tales from the pitch: Some observations on the gendered dynamics of English Collegiate Soccer. *Journal for the Study of Sports and Athletes in Education, 2*(2), 239–262. |
| Manual | Harris, K., & Adams, A. (2016). Power and discourse in the politics of evidence in sport for development. *Sport Management Review, 19*(2), 97–106. |
| Manual | Hassan, D. (2016). Surveillance by proxy: Sport and security in a modern age. *American Behavioral Scientist, 60*(9), 1043–1056. |
| Manual | Hodge, S., Burden, J., Robinson, L., & Bennett, R. (2008). Theorizing on the stereotyping of black male student-athletes: Issues and implications*. Journal for the Study of Sports and Athletes in Education, 2*(2), 203–226. |
| Manual | Hoeber, L. (2007). Exploring the gaps between meanings and practices of gender equity in a sport organization. *Gender, Work & Organization, 14*(3), 259–280. |
| Manual | Hoeber, L., & Kerwin, S. (2013). Exploring the experiences of female sport fans: A collaborative self-ethnography. *Sport Management Review, 16*(3), 326–336. |
| Manual | Hoffman, J. (2010). The dilemma of the senior woman administrator role in intercollegiate athletics. *Journal of Issues in Intercollegiate Athletics, 3*, 53–75. |
| Manual | Hovden, J. (1999). Is it worth the price? Women’s involvement in leadership and coaching in sport organizations in Norway. *Women in Sport & Physical Activity Journal, 8*(1), 23. |
| Manual | Hovden, J. (2015). Assessing the sociology of sport: On sport organizations and neoliberal discourses. *International Review for the Sociology of Sport, 50*(4–5), 472–476. |
| Manual | Hoye, R. (2006). Sports betting policy and product fees: Implications for Australian sports organisations. *Annals of Leisure Research, 9*(3–4), 155–172. |
| Manual | Hu, X. (Richard), & Henry, I. (2017). Reform and maintenance of Juguo Tizhi: Governmental management discourse of Chinese elite sport. *European Sport Management Quarterly, 17*(4), 531–553. |
| Manual | Hylton, K. (2010). How a turn to critical race theory can contribute to our understanding of ‘race’, racism and anti-racism in sport. *International Review for the Sociology of Sport, 45*(3), 335–354. |
| Manual | Knapp, B. A. (2015). Garters on the gridiron: A critical reading of the lingerie football league. *International Review for the Sociology of Sport, 50*(2), 141–160. |
| Manual | Knijnik, J., & Spaaij, R. (2017). No harmony: Football fandom and everyday multiculturalism in Western Sydney. *Journal of Intercultural Studies, 38*(1), 36–53. |
| Manual | Knoppers, A. (2011). Giving meaning to sport involvement in managerial work. *Gender, Work & Organization, 18*(s1), e1–e22. |
| Manual | Knoppers, A., & Anthonissen, A. (2005). Male athletic and managerial masculinities: Congruencies in discursive practices? *Journal of Gender Studies, 14*(2), 123–135. |
| Manual | Kochanek, J., & Erickson, K. (2019). Interrogating positive youth development through sport using Critical Race Theory. *Quest*, 1–17. |
| Manual | Levermore, R. (2011). Sport-for-Development and the 2010 Football World Cup. *Geography Compass, 5*(12), 886–897. |
| Manual | Lusted, J., & Fielding-Lloyd, B. (2017). The limited development of English women’s recreational cricket: A critique of the liberal “absorption” approach to gender equality. *Managing Sport and Leisure, 22*(1), 54–69. |
| Manual | Maguire, J., Barnard, S., Butler, K., & Golding, P. (2008). ‘Celebrate humanity’ or ‘consumers?’: A critical evaluation of a brand in motion. *Social Identities, 14*(1), 63–76. |
| Manual | Martin, A., & McDonald, M. G. (2012). Covering women’s sport? An analysis of Sports Illustrated covers from 1987-2009 and ESPN The Magazine covers from 1998-2009. *Graduate Journal of Sport, Exercise & Physical Education Research, 1*, 81-97. |
| Manual | Masucci, M., & Butryn, T. M. (2013). Writing about fighting: A critical content analysis of newspaper coverage of The Ultimate Fighting Championship from 1993-2006*. Journal of Sports Media, 8*(1), 19–44. |
| Manual | Mauro, M. (2017). Inclusive sport or institutional discrimination? New FIFA regulations, organized football and migrant youth in Italy. *Sport in Society, 20*(7), 833–847. |
| Manual | McDonald, M. G. (2017). Travelling discourses of Title IX: Gender and sport for development in an era of securitised interests. *Third World Thematics: A TWQ Journal, 2*(1), 37–53. |
| Manual | McDowell, J., & Carter-Francique, A. (2017). An intersectional analysis of the workplace experiences of African American female Athletic Directors. *Sex Roles, 77*(5–6), 393–408. |
| Manual | McGillivray, D., McPherson, G., & Misener, L. (2018). Major sporting events and geographies of disability. *Urban Geography, 39*(3), 329–344. |
| Manual | Misener, L. (2013). A media frames analysis of the legacy discourse for the 2010 Winter Paralympic Games. *Communication & Sport, 1*(4), 342–364. |
| Manual | Oxford, S., & Spaaij, R. (2017). Critical pedagogy and power relations in sport for development and peace: Lessons from Colombia. *Third World Thematics: A TWQ Journal, 2*(1), 102–116. |
| Manual | Pape, M. (2020). Gender segregation and trajectories of organizational change: The underrepresentation of women in sports leadership. *Gender & Society, 34*(1), 81–105. |
| Manual | Paton, K., Mooney, G., & McKee, K. (2012). Class, citizenship and regeneration: Glasgow and the Commonwealth Games 2014. *Antipode, 44*(4), 1470–1489. |
| Manual | Peers, D. (2009). (Dis)empowering Paralympic histories: Absent athletes and disabling discourses. *Disability & Society, 24*(5), 653–665. |
| Manual | Phillips, R. J. (2018). An Inquiry into the political economy of Hockey Night in Canada: Critically assessing issues of ownership, advertising, and gendered audiences. *Canadian Journal of Communication, 43*(2), 203–220. |
| Manual | Postlethwaite, V., Kohe, G. Z., & Molnar, G. (2018). Inspiring a generation: An examination of stakeholder relations in the context of London 2012 Olympics and Paralympics educational programmes. *Managing Sport and Leisure, 23*(4–6), 391–407. |
| Manual | Pringle, R. (2001). Examining the justifications for government investment in high performance sport: A critical review essay. *Annals of Leisure Research, 4*(1), 58–75. |
| Manual | Purdue, David E.J., & Howe, P. D. (2012). Empower, inspire, achieve: (Dis)empowerment and the Paralympic Games. *Disability & Society, 27*(7), 903–916. |
| Manual | Purdue, D.E.J., & Howe, P. D. (2012). See the sport, not the disability: Exploring the Paralympic paradox. *Qualitative Research in Sport, Exercise and Health, 4*(2), 189–205. |
| Manual | Rauscher, L., Kauer, K., & Wilson, B. D. M. (2013). The healthy body paradox: Organizational and interactional influences on preadolescent girls’ body image in Los Angeles. *Gender & Society, 27*(2), 208–230. |
| Manual | Rowe, D. (2009). Power trip: Sport and media. *Int. J. Sport Management and Marketing,* *6*(2), 150–166. |
| Manual | Rowe, D., & Gilmour, C. (2010). Sport, media, and consumption in Asia: A merchandised milieu. *American Behavioral Scientist, 53*(10), 1530–1548. |
| Manual | Ryan, I., & Dickson, G. (2018). The invisible norm: An exploration of the intersections of sport, gender and leadership. *Leadership, 14*(3), 329–346. |
| Manual | Samatas, M. (2011). Surveillance in Athens 2004 and Beijing 2008: A comparison of the Olympic surveillance modalities and legacies in two different Olympic host regimes. *Urban Studies, 48*(15), 3347–3366. |
| Manual | Sant, S.-L., & Mason, D. S. (2015). Framing event legacy in a prospective host city: Managing Vancouver’s Olympic bid. *Journal of Sport Management, 29*(1), 42–56. |
| Manual | Sartore, M., & Cunningham, G. (2010). The lesbian label as a component of women’s stigmatization in sport organizations: An exploration of two health and kinesiology departments. *Journal of Sport Management, 24*(5), 481–501. |
| Manual | Staurowsky, E. J., & Weight, E. A. (2014). Title IX literacy among NCAA administrator and coaches: A critical communications approach. *The Journal of Intercollegiate Sport, 15*(4), 1-29. |
| Manual | Schull, V., Shaw, S., & Kihl, L. A. (2013). “If a woman came in … She would have been eaten up alive”: Analyzing gendered political processes in the search for an athletic director. *Gender & Society, 27*(1), 56–81. |
| Manual | Scott, D. K. (1997). Managing organizational culture in intercollegiate athletic organizations. *Quest, 49*(4), 403–415. |
| Manual | Shaw, S. (2006). Scratching the back of “Mr X”: Analyzing gendered social processes in sport organizations. *Journal of Sport Management, 20*(4), 510–534. |
| Manual | Shaw, S., & Hoeber, L. (2003). “A strong man is direct and a direct woman is a bitch”: Gendered discourses and their influence on employment roles in sports organizations. *Journal of Sport Management, 17*(4), 347. |
| Manual | Shaw, S., & Penney, D. (2003). Gender equity policies in national governing bodies: An oxymoron or a vehicle for change? *European Sport Management Quarterly, 3*(2), 78–102. |
| Manual | Sibson, R. (2010). “I was banging my head against a brick wall”: Exclusionary power and the gendering of sport organizations. *Journal of Sport Management, 24*(4), 379–399. |
| Manual | Spaaij, R. (2012). Beyond the playing field: Experiences of sport, social capital, and integration among Somalis in Australia. *Ethnic and Racial Studies, 35*(9), 1519–1538. |
| Manual | Spaaij, R., Magee, J., Farquharson, K., Gorman, S., Jeanes, R., Lusher, D., & Storr, R. (2018). Diversity work in community sport organizations: Commitment, resistance and institutional change. *International Review for the Sociology of Sport, 53*(3), 278–295. |
| Manual | Spaaij, R., Magee, J., & Jeanes, R. (2013). Urban youth, worklessness and sport: A comparison of sports-based employability programmes in Rotterdam and Stoke-on-Trent. *Urban Studies, 50*(8), |
| Manual | Thorpe, H., & Chawansky, M. (2017). The gendered experiences of women staff and volunteers in sport for development organizations: The case of transmigrant workers of Skateistan. *Journal of Sport Management, 31*(6), 546–561. |
| Manual | Toffoletti, K. (2017). Sexy women sports fans: Femininity, sexuality, and the global sport spectacle. *Feminist Media Studies, 17*(3), 457–472. |
| Manual | Van den Broucke, S., & Gama Gato, L. (2018). Contesting the brand: A media analysis of the image of Rio de Janeiro as host of the 2016 Summer Olympics in Dutch language newspapers. *European Journal for Sport and Society, 15*(3), 268–287. |
| Manual | Veri, M. J. (2006). Etched impressions: Student writing as engaged pedagogy in the graduate sport management classroom. *Quest, 58*(4), 443–464. |
| Manual | Wagner, U., & Pedersen, K. M. (2014). The IOC and the doping issue—An institutional discursive approach to organizational identity construction. *Sport Management Review, 17*(2), 160–173. |
| Manual | Walker, N. A., & Melton, E. N. (2015). The tipping point: The intersection of race, gender, and sexual orientation in intercollegiate sports. *Journal of Sport Management, 29*(3), 257–271. |
| Manual | Walker, N. A., & Sartore-Baldwin, M. L. (2013). Hegemonic masculinity and theinstitutionalized bias toward women in men’s collegiate basketball: What do wen think? *Journal of Sport Management, 27*(4), 303–315. |
| Manual | Walsh, A. J., & Giulianotti, R. (2001). This sporting mammon: A normative critique of the commodification of sport. *Journal of the Philosophy of Sport, 28*(1), 53–77. |
| Manual | Wiest, A., & King-White, R. (2013). Selling out (in) sport management: Practically evaluating the state of the American (Sporting) Union. *Sport, Education and Society, 18*(2), 200–221. |
| Manual | Wolter, S. (2015). A Critical Discourse Analysis of espnW: Divergent dialogues and postfeminist conceptions of female fans and female athletes. *International Journal of Sport Communication, 8*(3), 345–370. |
| Manual | Wood, Z. C., & Garn, A. C. (2016). University intramural sport administrators’ perceptions of gender modifications in intramural coed flag football. *Sport, Education and Society, 21*(7), 1036–1052. |
| Manual | Zagacki, K. S., & Grano, D. (2005). Radio sports talk and the fantasies of sport. *Critical Studies in Media Communication, 22*(1), 45–63. |
| Manual | Zakus, D. H., & Skinner, J. (2008). Modelling organizational change in the International Olympic Committee. *European Sport Management Quarterly, 8*(4), 421–442. |
| Manual | Ziakas, V. (2015). For the benefit of all? Developing a critical perspective in mega-event leverage. *Leisure Studies, 34*(6), 689–702. |
| Manual | Zipp, S., Smith, T., & Darnell, S. (2019). Development, gender and sport: Theorizing a feminist practice of the capabilities approach in sport for development. *Journal of Sport Management, 33*(5), 440–449. |
